# Ventral tegmental area dopamine and GABA neurons: Physiological properties and expression of mRNA for endocannabinoid biosynthetic elements

**DOI:** 10.1038/srep16176

**Published:** 2015-11-10

**Authors:** Collin B. Merrill, Lindsey N. Friend, Scott T. Newton, Zachary H. Hopkins, Jeffrey G. Edwards

**Affiliations:** 1 Brigham Young University Department of Physiology and Developmental Biology Provo, UT 84602 USA; 2 Brigham Young University Neuroscience Center Provo, UT 84602 USA

## Abstract

The ventral tegmental area (VTA) is involved in adaptive reward and motivation processing and is composed of dopamine (DA) and GABA neurons. Defining the elements regulating activity and synaptic plasticity of these cells is critical to understanding mechanisms of reward and addiction. While endocannabinoids (eCBs) that potentially contribute to addiction are known to be involved in synaptic plasticity mechanisms in the VTA, where they are produced is poorly understood. In this study, DA and GABAergic cells were identified using electrophysiology, cellular markers, and a transgenic mouse model that specifically labels GABA cells. Using single-cell RT-qPCR and immunohistochemistry, we investigated mRNA and proteins involved in eCB signaling such as diacylglycerol lipase α, N-acyl-phosphatidylethanolamine-specific phospholipase D, and 12-lipoxygenase, as well as type I metabotropic glutamate receptors (mGluRs). Our results demonstrate the first molecular evidence of colocalization of eCB biosynthetic enzyme and type I mGluR mRNA in VTA neurons. Further, these data reveal higher expression of mGluR1 in DA neurons, suggesting potential differences in eCB synthesis between DA and GABA neurons. These data collectively suggest that VTA GABAergic and DAergic cells have the potential to produce various eCBs implicated in altering neuronal activity or plasticity in adaptive motivational reward or addiction.

The mesocorticolimbic circuit attaches salience to novel rewarding stimuli, allowing adaptive reward and motivational processing. Reward stimuli are processed by increased dopamine (DA) neuron activation in the ventral tegmental area (VTA), causing DA release to downstream targets, primarily in the nucleus accumbens (NAc) (For review, see^1^). Modulation of DA release can alter reward processing and motivational behavior, such as in drug abuse where extreme alterations in DA release can induce compulsive drug-seeking behavior that is a hallmark of addiction. Therefore, modulation of DA release from the VTA represents a key mechanism in the development of addiction.

Several sources of potential DA modulation are present within the mesocorticolimbic circuit. VTA GABA cells, which innervate and inhibit DA cells, can modulate DA cell activity^2^,^3^, subsequently modifying DA release. For example, decreased GABA activity induces disinhibition of DA neurons and increases DA levels^4^,^5^,^6^. Direct VTA GABA projections into the NAc are also involved in DAergic signaling, reward, and associative learning^3^,^7^,^8^ and represent another potential source of DA modulation. DA and GABA neurons compose the most numerous cells within the VTA, with recently-identified glutamate neurons forming a small percentage of total neurons^9^. Because the role of local glutamatergic neurons in the VTA is unclear and they are a minority cell type, we focused our studies on the major VTA cell types, DA and GABA neurons.

Within the VTA, long-term synaptic plasticity is the likely cellular correlate mediating modulation of DA signaling that underlies the learned behavioral response or addictive component of reward. Some forms of long-term synaptic plasticity in the VTA are mediated by endocannabinoids (eCBs), such as eCB-dependent LTD^10^,^11^,^12^,^13^,^14^,^15^. This eCB-dependent plasticity is most often mediated by activation of postsynaptic type I metabotropic glutamate receptors (mGluRs), resulting in eCB synthesis and retrograde activation of eCB receptors such as cannabinoid receptor 1 (CB1)^16^,^17^,^18^. Given the large number of glutamatergic afferents to the VTA^19^ and the presence of local glutamatergic neurons^9^, as well as GABAergic input, eCB-mediated synaptic plasticity potentially occurs at multiple synapses within the region. Indeed, electrophysiological data suggest eCBs involved in synaptic plasticity are likely produced at glutamatergic synapses onto DAergic cells in the VTA^10^,^12^,^13^. However, molecular evidence for the expression of eCB biosynthetic enzymes^14^ is sparse, especially regarding eCB production within GABA neurons. It was previously considered that only principal cells such as DA cells, but not GABA cells, synthesize eCBs that modify synaptic signaling in the brain. However, recent evidence has demonstrated this is a false notion. For example, hippocampal GABA neurons do indeed express eCB-producing enzymes^20^,^21^, and in fact, these GABA cells directly modulate synaptic plasticity at the physiological level^22^,^23^. Importantly though, not all hippocampal GABA cells expressed mRNA for eCB biosynthetic enzymes, suggesting subtype-specific, heterogeneous expression of these enzymes^20^,^21^. Given the importance of GABAergic modulation of DA release, localizing eCB synthetic machinery within VTA neuron types is critical, particularly in GABAergic cells where data is lacking. As evidence of eCB biosynthetic enzymes and type I mGluR co-expression needed for eCB production within VTA DA or GABA neurons is limited, we examined their expression. We hypothesized that in addition to DA cells, that some populations of GABA cells expressed eCB and type I mGluRs, therefore potentially playing a role in VTA function of reward by modulating both cell types via eCB activity.

In order to distinguish between DA and GABA cells in the VTA several criteria are traditionally employed; most notably, the presence of electrophysiologically recorded I_h_ currents or sag potentials in DA neurons was a key discriminator between DA and GABA cell types^24^. However, several recent reports have demonstrated considerable overlap in physiology between DA and GABA neurons^25^,^26^,^27^, suggesting that traditional physiological identifiers are not unique to distinct neuron types. As correct neuronal identification was a critical factor in our study, and because the accuracy of physiological differentiation has been debated in the past, we examined physiological criteria including sag potential and firing frequency to determine whether they could assist in discriminating between DA and GABA cells. In addition, because we employed alternative identification methods such as RT-qPCR and a GFP-GAD67 mouse line^28^, which allow genetic positive identification of GABA neurons, we could also examine whether physiology is a good discriminator of cell identity. The use of a transgenic mouse model also allowed comparison of GABA cell physiological profiles between rats and mice, as well as provide support for our identification criteria using gene expression experiments. We anticipated that sag potentials would be among the better discriminators of cell types in this study.

Using real-time quantitative PCR (RT-qPCR) and immunohistochemistry, we characterized and examined VTA neurons for the enzymes that produce endocannabinoids and eicosanoids, namely diacylglycerol lipase α (DAGLα), 12-lipoxygenase (12-LO), and N-acyl-phosphatidylethanolamine-specific phospholipase D (NAPE-PLD), which synthesize 2-arachidonylglycerol (2-AG), 12-(S)-hydroperoxyeicosa-5Z,8Z,10E,14Z-tetraenoic acid (12-HPETE) and anandamide^29^,^30^,^31^,^32^, respectively. We also examined the expression of mGluR1 and mGluR5. We hypothesized that type I mGluRs would be co-expressed with eCB-related signaling components in at least some VTA neuron populations, with the potential for differential expression. Our data demonstrate that eCB biosynthetic enzymes and type I mGluRs are indeed co-expressed in VTA DA and many GABA cells, suggesting eCB production can occur within either cell type. Therefore, eCB-mediated processes such as synaptic plasticity could be induced at synapses of either VTA cell type. eCB-dependent processes are critical to understand because drugs of abuse acting in the VTA can alter or occlude these processes^33^,^34^,^35^,^36^,^37^, which may underlie altered reward processing leading to addiction.

## Results

Because little is known about the eCB system within VTA GABAergic neurons and their role in adaptive reward processes, we particularly focused on GABA neurons in this study. We extracted single cells from rat and mouse VTA and analyzed gene expression using RT-qPCR. Cells were identified based on cellular markers. We confirmed DAergic cell identity by positive expression of either tyrosine hydroxylase (TH) or DA transporter (DAT) mRNA. GABAergic neuron identity was confirmed by expression of either GAD65 or GAD67 mRNA. In rat cells, 84.6% of DA neurons expressed TH, with 63.6% of these TH positive cells co-expressing DAT and the remainder expressed DAT only. It is noteworthy that GAD65 was also expressed in 38.5% of DA neurons. Of the DA neurons that expressed GAD65, 100% co-expressed TH only and 60% co-expressed TH and DAT. We did not detect GAD67 co-expression with TH or DAT. Following these criteria, of 74 neurons extracted from rat brain slices, 16 were identified as DAergic, 12 were identified as GABAergic, 38 were unable to be classified based on the cell marker gene expression, and 8 were failures.

To examine mouse VTA neurons, we employed a mouse line with targeted knock-in of GFP in GAD67-expressing cells. Using GAD67-GFP allowed us to positively confirm a cell genetically as GABAergic rather than rely solely on cellular markers. We extracted 12 GFP-positive GABAergic cells and 18 non-GFP cells. GABAergic identity was confirmed by visual observation of GFP in the recording pipette and positive GAD67 mRNA expression. GAD67 mRNA was observed in 75% of GFP-expressing cells, indicating a 75% positive detection rate for our PCR methods. Neither TH nor DAT were ever detected in the GAD67-GFP cells we examined. These criteria allowed unequivocal identification of mouse GABAergic VTA neurons. Non-GFP cells were confirmed as DAergic by expression of TH or DAT mRNA. In addition, twenty GFP-positive, cytosol-only samples were also extracted as a control to verify positive expression of mRNA rather than nuclear DNA from GABA cells.

**Figure 1:**
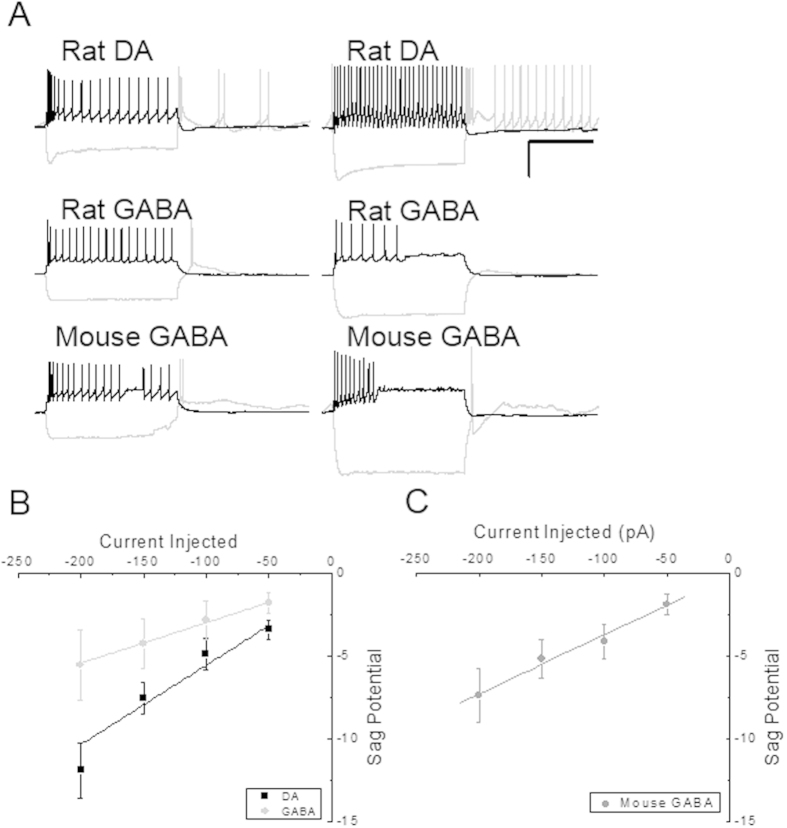
Electrophysiological properties of rat and mouse DA and GABA cells. (**A**) Selected examples of electrophysiological profiles of VTA dopaminergic and GABAergic neurons. Scale bars represent 35 mV and 500 msec for all traces. Positive current injection was either 100 or 150 pA in each, and hyperpolarizing current injected was −200 pA. DA neurons were identified by expression of TH or DAT and GABA neurons were identified by expression of GAD65 or GAD67. (**B**) Sag potential amplitude increases in a linear manner with increasing negative current injection, and while overlapping in some cases, differs significantly (p < 0.003, ANCOVA) between rat DA (black) and GABA (gray) neurons. (**C**) Sag potential amplitude increases in a linear manner with increasing negative current injection in mouse GABA neurons, which were also not significantly different from rat GABA cells (p = 0.85, ANCOVA), but were significantly different from rat DA neurons (p < 0.01, ANCOVA).

To further examine neuron identity, we obtained electrophysiological profiles of VTA neurons to determine whether these profiles are reliable criteria to distinguish between DA and GABA cells, as has been recently debated. We analyzed action potential frequency and pattern at 150 pA injected current and sag potential amplitude and rebound spiking at −200 pA injected current in all cells. Collectively, many electrophysiological properties were similar between DA and GABA neurons, including action potential pattern and firing frequency. Rat DAergic neurons fired in two patterns: regular (n = 7) or rapidly adapting (n = 3). Evoked action potential frequency varied from 16.7–32.6 Hz (25.9 ± 2.0 Hz average) in regular spiking cells, and from 28.8–93.6 Hz (66.1 ± 14.8 Hz average) in rapidly adapting cells. Rapidly adapting cells fired 3–7 action potentials before adapting. TH-positive/GAD65-positive DA neurons all fired in a regular pattern. After a hyperpolarizing current injection of −200 pA, DA neurons generally fired rebound action potentials in a regular train or a burst of approximately five action potentials (Fig. 1A). GABA neurons fired in a regular (n = 4) or adapting (n = 7) pattern, with regular spiking cells ranging from 13.9–28.3 Hz (19.7 ± 3.1 Hz average) and rapidly adapting cells ranging from 12.2–54.2 Hz (38.4 ± 5.8 Hz average). Also, in contrast to DA cells, rat GABAergic neurons generated either zero, one, or two rebound action potentials after hyperpolarization with −200 pA current injection (see Fig. 1A).

Sag potentials from all DA neurons were similar, ranging from −9.6 to −28.8 mV (−19.9 ± 1.9 mV average) during −200 pA hyperpolarization. Sag potential amplitude of DA neurons increased with increased negative current injection. Regular and adapting GABA neurons displayed smaller sag potentials ranging from −1.9 to −9.8 mV (−4.6 ± 1.7 mV average) in response to −200 pA current injection, with an increase in sag potential amplitude with increased current injection (Fig. 1B). GABA cells (n = 11) sag potential amplitude was significantly smaller (p < 0.001, t-test) and smaller rate of sag potential increase (Fig. 1B, p = 0.003, ANCOVA) than DA neurons (n = 10).

To provide a positive control comparator for electrophysiological profiles of rat GABAergic cells, we examined physiological profiles in genetically identified mouse GABAergic neurons. Patterns of evoked action potential firing fell into three groups: regular (n = 5), irregular (n = 4), and rapidly adapting (n = 10). Regular firing neurons ranged from 21.2 to 47.8 Hz (30.8 ± 4.7 Hz average), irregular firing neurons ranged from 11.8 to 34.7 Hz (22.6 ± 4.8 Hz average), and rapidly adapting neurons ranged from 6.5 to 99.4 Hz (54.6 ± 8.6 Hz average). Irregular spiking neurons generally fired several quick action potentials at the beginning of stimulation with subsequent action potentials at irregular intervals for the duration of stimulation. Mouse GABA neurons generally fired either zero, one, or two rebound action potentials following hyperpolarization with −200 pA current injection, identical to rat GABA cells. Further, these cells displayed sag potentials from −1.2 to −24.9 mV (−8.8 ± 1.7 mV average) during −200 pA hyperpolarizing current injection (Fig. 1C). Sag potential amplitude was very similar to cells classified as GABA cells in rats (p > 0.25, t-test), with similar rate of sag potential increase (Fig. 1C; p = 0.85, ANCOVA), but were statistically different from rat DA neurons for both sag potential amplitude (p < 0.05, t-test) and rate of sag potential increase (Fig. 1C; p < 0.01, ANCOVA). Electrophysiological parameters from mouse GAD67-GFP GABAergic cells were similar to those of neurons classified as GABAergic via RT-qPCR from rat VTA, supporting our PCR characterization of cell types. I_h_-related sag potentials and rebound potentials evoked by hyperpolarizing current injection, but not evoked action potential frequency, were the best discriminators between DA and GABA neurons. However, it should be noted that these data also illustrate some overlap in electrophysiological characteristics between VTA DA and GABA neurons, demonstrating these characteristics are not infallible in characterizing DA from GABA cells. Collectively, electrophysiology characteristics supported our classification by RT-qPCR and additionally we report a novel finding that rebound action potentials following hyperpolarization in DA neurons appears to be a potentially useful criterion to distinguish between cell types.

GABA neurons from various brain regions can often be categorized into subtypes based on expression of the calcium binding proteins parvalbumin (PV), calbindin (CB), and calretinin (CR), and the neuropeptide CCK, among others. To determine if neuron subtypes exist in the VTA, we tested rat VTA neurons for the presence of these targets. We detected PV, CB, CR, and CCK expression (see Table 1) with a large degree of colocalization within rat GABAergic neurons. Almost all CCK-positive GABA neurons co-expressed both PV and CB (n = 5, 83.3%). This high depress of co-expression was not noted in DA cells where expression of PV, CB, CR was more sparse with eight cells lacking expression of calcium binding protein mRNA. However, CCK expression was frequently observed in DA cells (see Table 1). These data demonstrate both that multiple calcium binding protein and CCK co-expression is prevalent in VTA GABA cells and that calcium binding protein expression occurs more frequently in GABAergic neurons than DAergic neurons. While GABA cells did not however, fall into previously categorized subtypes, as seen in other brain areas such as the hippocampus^20^, they did differ from DA cells again supporting PCR characterization.

**Figure 2:**
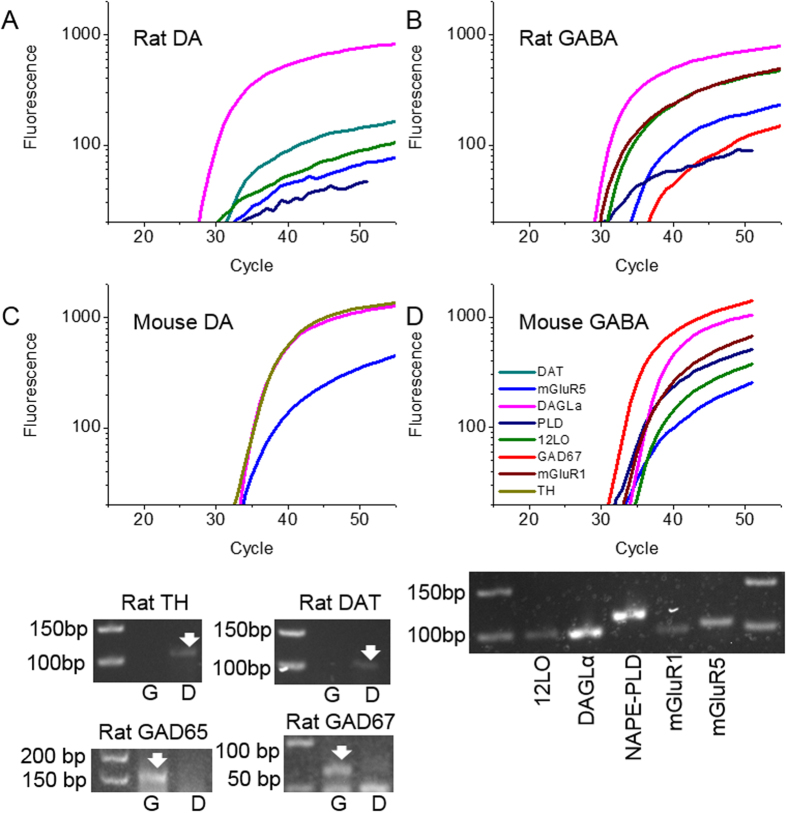
Gene expression profiles of ventral tegmental area dopaminergic and GABAergic neurons. (**A**) A representative rat DAergic cell expressing DAGLα, 12LO, DAT, mGluR5, and NAPE-PLD. (**B**) A representative rat GABAergic neuron expressing DAGLα, mGluR1, 12LO, NAPE-PLD, mGluR5, and GAD67. (**C**) A representative mouse DAergic neuron expressing TH, DAGLα, and mGluR5. (**D**) A representative mouse GABAergic neuron expressing GAD67, NAPE-PLD, mGluR1, mGluR5, DAGLα, and 12LO. Relative fluorescence was generated by FAM-TAMRA probes and graphed relative to the number of PCR cycles performed. Inset: Gel electrophoresis from the PCR reaction of a mouse GAD67-GFP neuron demonstrating the expression of 12LO (100 bp), DAGLα (100 bp), NAPE-PLD (104 bp), mGluR1 (78 bp), and mGluR5 (103 bp). Inset: Gel electrophoresis from a PCR reaction demonstrating cell-type specific expression of TH/DAT and GAD65/67 within rat DA (**D**) and GABA (**G**) neurons. Amplicon sizes for PCR products are TH: 107 bp; DAT: 101 bp; GAD65: 155 bp; GAD67: 73 bp.

Next, we examined the co-expression of mRNA coding for the eCB system components DAGLα, NAPE-PLD, 12LO, and mGluR1/5. Within identified rat DA and GABAergic cells, we detected expression of all of these targets (Fig. 2A,B, Table 2). Indeed, the expression of these between DA and GABA neurons is strikingly similar, however NAPE-PLD expression occurred more frequently in DAergic neurons (Table 2).

Next, to confirm our results in rat GABA cells, we examined expression of these targets in neurons genetically identified as GABAergic from GAD67-GFP mice. In 12 GABA neurons, we observed expression of all eCB enzymes and both type I mGluRs (Fig. 2D, Table 2). These expression patterns were very similar to our classified rat VTA GABAergic cells, confirming the data from rat VTA GABA cells was accurate. As an additional control, 20 cytosol-only GAD67-GFP samples devoid of cellular DNA were tested, confirming these samples expressed DAGLα (30.0%), NAPE-PLD (5.0%), 12LO (5.0%), mGluR1 (15.0%), and mGluR5 (55.0%), similar to rat and mouse single-cell samples, which verified our whole-cell data. These expression levels were slightly reduced compared to whole cell extractions, as would be expected since there is less starting material to isolate mRNA from.

Colocalization of eCB biosynthetic enzyme mRNA and type I mGluR mRNA occurred in both rat DA and GABA cells, with especially high co-expression of mGluR5 and DAGLα in GABA neurons (Fig. 2A,B, Table 2). Within GAD67-GFP-positive mouse whole-cell samples, mGluR1/5 co-localized with these enzymes as well (Fig. 2D, Table 2), consistent with expression patterns from rat VTA GABAergic neurons. Collectively, these data demonstrate that VTA DA and GABAergic neurons both express the cellular machinery necessary for eCB synthesis.

**Figure 3:**
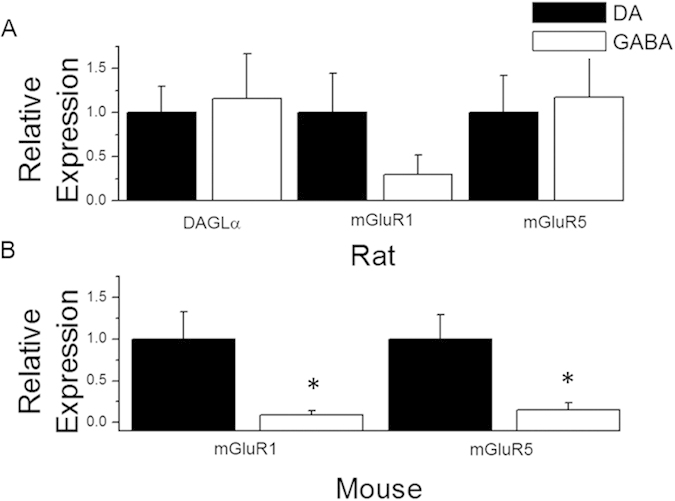
Quantification of mGluR1, mGluR5 and DAGLα mRNA in rat ventral tegmental area neurons. (**A**) Relative mRNA levels of DAGLα, mGluR1, and mGluR5 in rat ventral tegmental area neurons illustrate higher, but not significantly different levels of DAGLα and mGluR5 in VTA GABA cells with lower levels of mGluR1 in GABA cells. (**B**) Relative mRNA levels of mGluR1 and mGluR5 in mouse ventral tegmental area neurons illustrate mGluR1 and mGluR5 are expressed at a higher level in DA neurons (p = 0.05, mGluR1; p < 0.05, mGluR5; asterisk). Error bars represent SEM. Expression data was normalized to DA neurons in all quantification analyses.

We next quantified the expression levels of eCB biosynthetic enzyme and type I mGluR mRNA within VTA neurons. Within rat brain slices (Fig. 3A), DAGLα mRNA levels were not significantly different (p = 0.8) between GABA neurons (n = 6; 115%) and DA neurons (n = 7). Rat mGluR1 mRNA levels in GABA neurons (n = 4; 29%) were not significantly different from DA neurons (n = 3, p = 0.25, t-test), and mGluR5 expression in GABA neurons (n = 7; 117%) were not significantly different than in DA neurons (n = 5, p = 0.80, t-test). As the most intriguing differences in expression in rat neurons were between type I mGluRs in DA versus GABA cells, we subsequently examined mouse DA cells with a focus on type I mGluRs. mGluR1 expression in mouse GABA neurons (n = 5; 8%) was significantly lower than in DA neurons (n = 5, p < 0.05, t-test, Fig. 3B) and mGluR5 expression was also significantly reduced in GABA neurons (n = 5; 15%) compared to expression in DA neurons (n = 7, p = 0.03, t-test).

**Figure 4:**
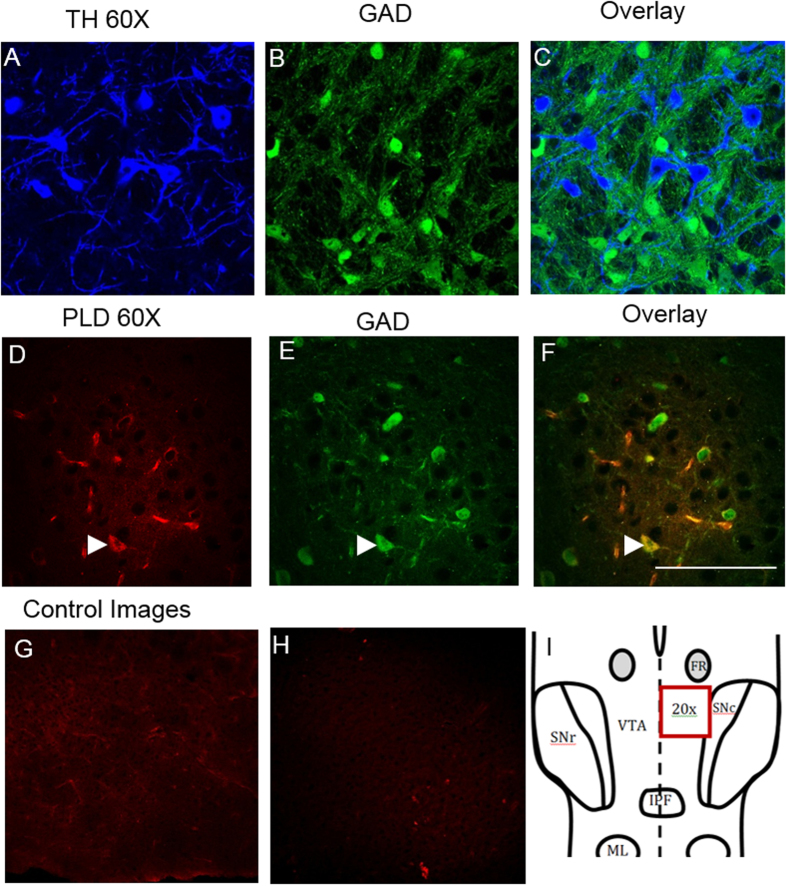
Immunolabeling of NAPE-PLD within mouse ventral tegmental area horizontal slices. TH immunoreactivity is distinct from GAD67-GFP positive neurons, demonstrating that identification of VTA neurons by these targets firmly establishes neuronal identity. Cytoplasmic protein expression of NAPE-PLD was noted clearly within many, but not all, GAD67-GFP VTA neurons. (**A**–**C**) TH and GAD67-GFP at 60× magnification demonstrate distinct VTA dopamine and GABA cells populations. Note that some non-labeled cells (non-TH/GAD67) noted were not quantified. (**D**–**F**) NAPE-PLD immunoreactivity at 60× magnification. Arrowheads identify positive immunolabeling of NAPE-PLD in GFP-positive GABA neurons. Scale bars: 100 μm. (**G**) Control image showing secondary antibody staining only. (**H**) Control image with preabsorption using a blocking peptide for DAGLα. Preabsorption and secondary antibody-only staining was done for NAPE-PLD and mGluR5 as well, with similar results (data not shown). (**I**) A schematic diagram of VTA showing area imaged in immunohistochemical experiments.

**Figure 5:**
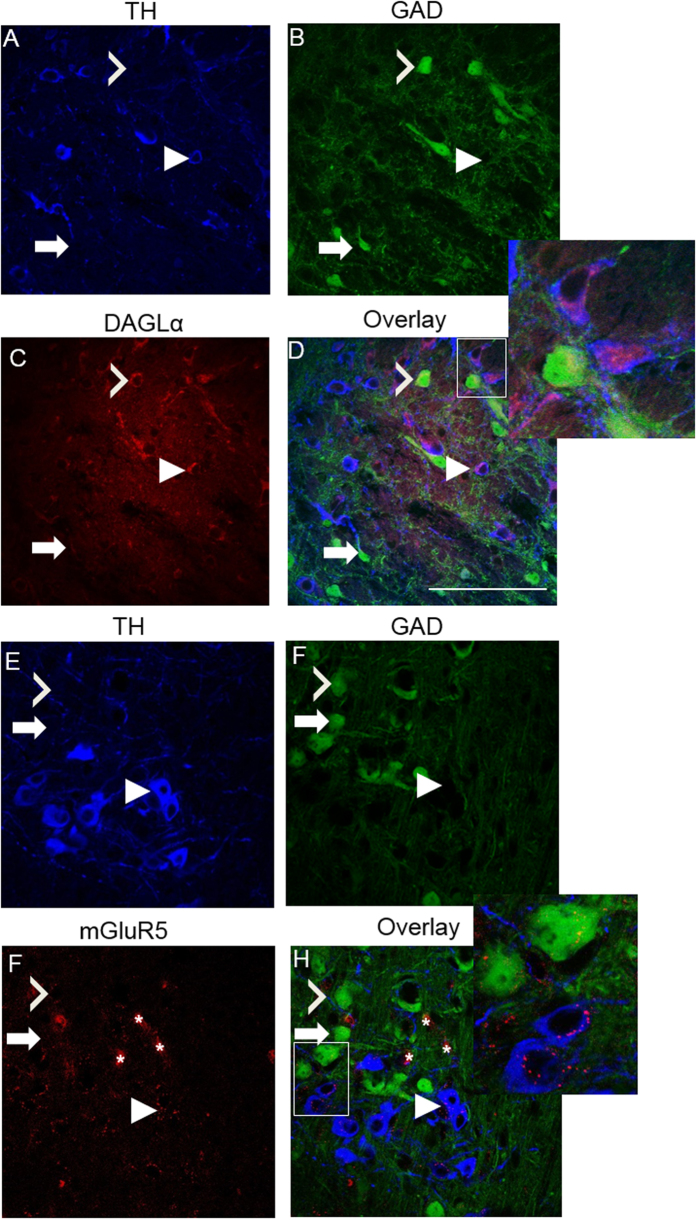
Triple immunolabeling of DAGLα and mGluR5 within VTA DAergic and GABAergic neurons. Immunofluorescence showing DAGLα and mGluR5 distribution in VTA neurons, with clear labeling in the cytosol of most DA and some GABA neurons. (**A**) TH immunolabeling, (**B**) GAD67-GFP labeling, (**C**) DAGLα immunolabeling, (**D**) Merged images. (**E**) TH immunolabeling, (**F**) GAD67-GFP labeling, (**G**) mGluR5 immunolabeling, (**H**) Merged images. All images were taken at 60× magnification and were acquired from slices taken from 2 animals. Closed arrowheads: TH-positive target-positive labeled neuron; open arrowheads: GAD67-GFP target-positive labeled GABA neuron; arrow: GFP-positive, target-negative neuron. Asterisk: Non-specific blood vessel labeling that occurred in mGluR5 immunostaining experiments. Scale bars: 100 μm.

Following RT-qPCR analysis, we performed immunohistochemical experiments using GAD67/GFP mice for a few important targets including DAGLα, NAPE-PLD, and mGluR5. A TH antibody was used to identify DA cells, while GFP identified GABA cells. First, GAD67 never co-localized with TH in VTA neurons (Fig. 4A–C), suggesting that the GAD67-GFP positive neurons we examined are indeed all GABAergic. Similarly, GAD67-TH co-expression never occurred in our RT-qPCR experiments. Positive TH labeling occurred in 69.4% of cells while GFP appeared in the remaining 30.6% of labeled neurons (average of 4 images at 20× magnification). We observed positive immunolabeling of NAPE-PLD, DAGLα, and mGluR5 within many, but not all, GFP labeled GABA cells. Low magnification images were used to assess positive immunolabeling in VTA neurons. NAPE-PLD co-localized with GAD67 in 56% of GFP-positive GABA neurons (Fig. 4D–F; n = 75). Next, in triple labeled slices, DAGLα co-localized with TH in 80.0% of cells (n = 85), and co-localized with GAD67 in 66% of GFP-positive cells (n = 68; Fig. 5A–D) and mGluR5 (Fig. 5E–H) was observed in 61% of TH-positive neurons (n = 21) and 55% of GAD67-GFP-positive neurons (n = 31). Collectively, percentages of expression from immunohistochemistry were very similar to expression noted in qPCR experiments, albeit at times with higher protein expression levels compared to mRNA expression levels, which would be expected based on occasional false negatives that commonly occur with single cell RT-qPCR.

## Conclusion

Modulation of DA transmission by eCBs, especially via synaptic plasticity, plays a role in long-term alterations associated with addiction^10^,^11^,^12^,^13^,^14^,^38^,^39^, but until now, the neuronal source of eCBs within the VTA was poorly understood. Additionally, GABAergic signaling by inhibitory cells has recently been demonstrated as critical to VTA function, both modulating DA levels^3^ and undergoing LTP_GABA_-type synaptic plasticity^36^,^37^,^40^,^41^, but evidence for their role in eCB modulation of DA signaling is very sparse. Our data demonstrate that both VTA DAergic and that some but not all GABAergic neurons co-express mRNA for enzymes and receptors involved in eCB signaling, suggesting that both these cell types can potentially play a role in eCB modulation of DA signaling via eCB-mediated activities such as synaptic plasticity.

It is important to note that when interpreting results regarding RT-qPCR, a lack of detection does not necessarily indicate absence of the tested target. For example, detecting GAD67 in 75% of GAD67/GFP-labeled cells indicates the rate of false negatives was approximately 25%, since all these cells express GAD67. Therefore, data and analyses discussed herein may underestimate actual mRNA expression levels, which is common for RT-qPCR experiments. In addition, we classified 38 cells as unidentified due to lack of detected TH/DAT and GAD65/67 expression likely due to false negatives. While these cells could not be categorized, they expressed mRNA for many eCB biosynthetic enzymes and type I mGluRs, and were likely DA, GABA, or glutamatergic cells. To help compensate for false negatives, we routinely performed RT-qPCR on an entire cell to maximize mRNA collected. By combining RT-qPCR and immunohistochemical data, we are confident that the positive expression of detected mRNA products we report within VTA neurons is accurate.

*Ex vivo*, there is no one reliable method other than positive genetic identification to determine neuron identity, so the combination of several methods such as physiology and gene expression is often used to characterize VTA neurons (for review, see^42^). For example, DA neurons generally fire at lower frequencies and display larger sag potentials during hyperpolarizing current injection^43^, though recent evidence suggests that distinct DA populations with faster firing frequencies are present^44^,^45^. In contrast, GABA neurons tend to fire at higher frequencies and display very small or absent I_h_-related sag potentials^26^,^27^,^46^,^47^,^48^, though this is not always the case^26^,^27^. Our data similarly demonstrate overlap in physiological properties between DA and GABA neurons, as suggested by others^26^,^27^. Therefore, physiological properties alone cannot be used exclusively to characterize VTA cell types, though others suggest spontaneous action potential duration may provide the most accurate identification^25^. In our current study, I_h_-related sag potentials and rebound spiking following hyperpolarization seem to be the best characteristics to distinguish VTA neuron identity, at least for the age of animal used in this study.

Concerning gene expression, while standard cellular markers are usually fairly reliable for classification, on occasion GAD65 does co-localize with TH^46^,^49^,^50^, especially in GABA-releasing DA neurons that project to lateral habenula^44^. Similarly, our data demonstrated GAD65 co-expression with TH in approximately over one-quarter of TH-positive cells tested cells by PCR. We never detected GAD67 co-expression with TH or DAT in rat GABA or mouse GAD67-GABA neurons by PCR or immunohistochemistry, as demonstrated in mouse by others^25^. Therefore, GAD67 is a good marker to identify VTA GABA neurons, making the GAD67-GFP mouse model appropriate for this study.

In the cerebral cortex, the presence of neuropeptides and calcium binding proteins can classify cells into subtypes, particularly GABA cells^51^. In VTA GABA neurons, previous studies have observed co-localization of CCK, PV, CB and CR with GAD65/67^28^,^49^,^52^,^53^. Our data was very similar in that there was a high degree of calcium-binding protein and CCK expression in GABA cells. Also in our study, three cells expressed CR with no colocalization of PV, CB, or CCK, which in the hippocampus and cortex is indicative of an interneuron-selective GABA cell that only innervates other GABA cells^54^,^55^. However, differentiation of GABA cells into distinct subtypes as demonstrated in the hippocampus and cortex^51^,^56^ was not possible using the targets we examined. This suggests that either distinct GABAergic subtypes are not as common in the VTA or that these subtypes, if present, must be classified using other criteria such as axon innervation pattern, projection target or other markers. Therefore, we do not know which GABA subtypes do or do not potentially produce eCBs. Our data demonstrate that co-expression of multiple calcium-binding proteins and neuropeptides within VTA GABA cells is common, unlike other brain regions where high degree of co-expression is less common.

Regarding DA cells, expression of TH with CCK, CB or CR is well-established^47^,^55^,^57^,^58^,^59^, as is some colocalization of TH with both CB and CR^60^. Importantly, PV expression has never been directly tested in VTA DA neurons. This study is the first to describe positive PV mRNA expression in some DA neurons. In summary, our data note a higher degree of co-expression of calcium binding protein and neuropeptide mRNA in GABA neurons compared to DA neurons, and that CCK co-expression with CR and PV within GABA cells may distinguish them from DA cells.

DAGLα synthesizes 2-AG via mGluR5 activation^18^. High levels of DAGLα are present at glutamatergic synapses in the majority of the brain such as the hippocampus, cortex, and cerebellum^10^,^29^,^61^,^62^. Further, DAGLα expression was identified in pyramidal neurons and distinct interneuron subtypes in the hippocampus^20^,^22^. However, DAGLα was not detected at symmetric GABA synapses within the hippocampus^63^, which coupled with previous observations of very low DAGLα expression in CR-positive neurons in the hippocampus^20^, suggests a lack of DAGLα expression by certain GABA neurons. As DAGLα was identified in DA and non-DA neurons within the VTA^14^ and is involved physiologically in synaptic plasticity at excitatory synapses onto DA cells^10^,^14^,^38^,^64^, it suggests both VTA GABA and DA cells may express DAGLα. Our RT-qPCR data indeed confirm DAGLα mRNA expression within positively identified VTA GABA neurons. Identification of similar levels of DAGLα expression in both DA and GABA cells suggests that 2-AG can be produced at multiple locations within the VTA and may allow modulation of DA transmission via 2-AG mediated plasticity at multiple synapses within the circuit.

NAPE-PLD produces anandamide, which binds multiple eCB receptors, including CB1. The expression of NAPE-PLD was previously observed within the VTA by *in situ* hybridization, but this expression was not evaluated within distinct neuronal populations^65^. The physiological effects of anandamide within the VTA have only been indirectly tested using CB1 or fatty acid amide hydrolase antagonists, and suggest anandamide is not involved in CB1-mediated synaptic plasticity at VTA inputs^13^,^38^,^64^,^66^. However, fatty acid amide hydrolase antagonists block some effects of nicotine and cocaine on medium spiny neurons of the NAc^67^, suggesting a role for anandamide in the mesocorticolimbic circuit. The RT-qPCR data presented here is the first demonstrating expression of NAPE-PLD mRNA within distinct VTA neuron populations, and the first description of NAPE-PLD mRNA or protein within VTA GABA neurons, which is similar to other GABAergic neuron populations in brain areas such as the hippocampus^20^,^21^.

Finally, 12LO activity is thought to play a role in the acute phase of addiction via alterations to glutamate activation of DA cells^68^,^69^. Further, 12-HPETE produced by 12LO is a TRPV1 agonist and is involved in TRPV1-mediated synaptic plasticity^23^,^70^. TRPV1 activation was observed to excite DA neurons^71^, which may also be caused by N-arachidonoyl-dopamine activation of TRPV1^72^ and may modulate opiate reward^73^. The expression of 12LO within the VTA suggests a possible role in VTA synaptic plasticity. This is the first observation of 12LO in both DA and GABA neurons, though at lower levels then NAPE-PLD and DAGLα. Taken together, while 12-LO is expressed by VTA neurons, the function of 12LO within the VTA remains unclear and requires further investigation.

Regarding eCB production, it is critical to understand expression patterns of type I mGluRs, as mGluRs are involved in eCB biosynthesis in many brain areas^16^,^17^. Both mGluR1 and mGluR5 are expressed within the VTA^74^. mGluR1 expression in VTA DA neurons is involved in several types of synaptic plasticity, particularly mGluR-LTD^75^,^76^,^77^. mGluR5 activation causes 2-AG synthesis^18^, and its activity increases DA levels in NAc and prefrontal cortex^78^,^79^,^80^, which is a key factor in cocaine sensitization^81^,^82^,^83^,^84^. Our data represent the first quantification of type I mGluR mRNA expression in VTA DA and GABA neurons by RT-qPCR and the first description of type I mGluR mRNA within VTA GABA neurons using immunohistochemistry. Differences noted in type I mGluR and eCB enzyme expression patterns within VTA neurons could correlate to functional differences. In addition, higher mGluR5 expression within mouse DA cells compared to GABA cells may suggest these cells are more likely to produce eCBs via mGluR5 activation, though we do not have a rationale to explain differences in mGluR5 expression between rat and mouse VTA neurons. Finally, immunohistochemical experiments demonstrated type I mGluR and eCB biosynthetic enzyme expression does not occur in all DA and GABA cells, suggesting possible differential expression based on subpopulations of each neuron type.

In summary, co-expression of type I mGluRs and eCB biosynthetic enzymes in VTA GABA and DA cells suggest that certain populations of both cell types have the capacity to produce eCBs and thus be involved in eCB-dependent synaptic plasticity or modulation of DA signaling during adaptive reward, motivational processing or addiction.

## Methods

### Slice Preparation

All experiments were performed in accordance with Institutional Animal Care and Use Committee protocols and followed NIH guidelines for the care and use of laboratory animals. IACUC protocols for all experimental protocols for both rats and mice were approved by the Brigham Young University Institutional Animal Care and Use Committee, Animal Welfare Assurance Number A3783-01. Male Sprague-Dawley rats (16–28 days old) were used for RT-qPCR experiments and male GAD67-GFP (16–28 day old) mice^28^ were used for RT-qPCR and immunohistochemistry experiments. A total of 18 rats and 20 mice were used for all experiments. All animals were anesthetized using isoflurane and decapitated using a rodent guillotine. The brain was rapidly removed, sectioned into 300 μm (mouse) or 400 μm (rat) thick horizontal slices, and stored at 34 °C on a net in artificial cerebrospinal fluid containing (in mM) 220 sucrose, 0.2 CaCl_2_, 3 KCl, 1.25 NaH_2_PO4, 25 NaHCO_3_, 12 MgSO_4_, and 10 glucose, saturated with 95% O_2_/5% CO_2_ (pH 7.4).

### Electrophysiological Recordings and Extraction

Slices were transferred to a submerged recording chamber and bathed in filtered, oxygenated, artificial cerebrospinal fluid at a flow rate of 2–3 mL/min. Ventral tegmental area neurons were visually selected using infrared or fluorescence optics via a CCD camera and monitor connected to an Olympus BX51WI microscope with a 40× water immersion objective. As a note, many cells were extracted from the anterior VTA, as a higher percentage of GABA cells were located within that region as noted in slices from GAD-GFP mice. Upon selection, each cell was patched with a borosilicate glass pipette filled with approximately 3 μL filtered internal solution composed of (in mM) 117 potassium gluconate, 2.8 NaCl, 20 HEPES, 5 MgCl2, 2 ATP-Na, 0.3 GTP-Na, and 0.6 μM EGTA-K (pH 7.28, 275–285 mOsm). Patch pipette resistance was 1.5–3 MΩ. Cells were held at −65 mV prior to recording. Spiking patterns were evoked in whole cell current clamp configuration, with a stimulus protocol consisting of a 50 msec baseline recording, a one second stimulation step, and a final 950 msec baseline recording. No current was injected during the baseline portions of the protocol. During the stimulation portion of the protocol, current was injected from −300 to 600 pA in 50 pA steps to elicit I_h_-related sag potentials and evoke action potential firing. Spontaneous firing activity was not monitored, only evoked action potential frequency. Electrophysiological data were recorded with a Multiclamp 700B amplifier (Molecular Devices, Sunnyvale, CA). Signals were filtered at 4 kHz and digitized with an Axon 1440A digitizer (Molecular Devices) connected to a Dell personal computer with pClamp 10.3 software (Molecular Devices). Electrophysiological data were analyzed using Clampfit10.3 software (Molecular Devices). Any cell with a resting potential more positive than −35 mV was excluded from electrophysiological analysis as they were not considered reliable. Sag potential amplitude and firing frequencies were analyzed by Student’s T-test.

Recorded neurons were extracted immediately as described^20^. Briefly, we extracted single neurons following electrophysiological recordings using the same pipette, using a slightly modified extraction method for both cytosol and whole cell samples^85^,^86^. After recordings, negative pressure was applied to each cell with a 10 mL syringe attached to the recording electrode. A giga-ohm seal was maintained throughout the extraction and the pipette was carefully withdrawn from the slice, keeping the cell attached. Once free of the slice, we ensured the cell was visually free from debris, and the extracted cell was transferred immediately into chilled reverse transcription mixture by breaking the tip of the electrode into an ultracentrifugation tube and expelling the contents with positive pressure^87^. To ensure cellular DNA did not cause false positive results, some cytosol-only control samples were also extracted. For these samples, the same 1.5–3 MΩ electrodes were used and cytosol was aspirated by gentle suction under visual observation^86^,^87^. Afterwards, the pressure was released and the pipette was carefully withdrawn without suction, leaving the cell and nucleus in the slice. In GAD67/GFP-positive mice, to ensure collection of the correct cell, the presence of cytosol was visually verified by observation of GFP in the pipette tip. The pipette was then removed from the slice and the cytosol transferred into chilled reverse transcription solution as described above. All single cells and cytosol-only samples were processed by reverse transcription reaction within two hours of extraction. An artificial cerebrospinal fluid control sample was extracted from every slice. The same type of electrode was first placed in the slice with no pressure, removed, and then fluid was aspirated by negative pressure generated by a 10 mL syringe attached to the recording pipette, while just above the slice. As background mRNA can be present in the ACSF as a result of the slicing procedure due to damaged cells releasing their contents, these controls were completed to ensure that any contaminating mRNA identified in these artificial cerebrospinal fluid controls that would result in false positives were eliminated from single cell analysis of cells taken from those same slices^88^. All samples were processed in the same manner.

### Reverse Transcription Reaction

The reverse transcription reaction was performed with iScript cDNA Synthesis Kit (BioRad), following the prescribed protocol, with a final reaction mixture of 12 μL. This mixture was then cycled in a C1000 Thermocycler (BioRad) under the following conditions: 25.0 °C for 8 minutes, 42.0 °C for 60 minutes, and 70 °C for 15 minutes.

For primer optimization (see below for more details) a cDNA library was created by reverse transcription of mRNA from homogenized brain tissue. Homogenates were obtained from rats and mice. Homogenization and mRNA extraction were performed using TriZol reagent (Invitrogen), according to its published protocol, followed by mRNA conversion to cDNA using iScript cDNA synthesis kit (BioRad), according to its published protocol.

### Primer Design, Verification, and Optimization

All primers for tested targets were designed using Vector NTI software (Invitrogen) and PrimerExpress software (Applied Biosystems Inc.), using identical parameters (T_m_, GC content, minimum primer length) for each primer set (for rat primer/probe sequences, see^20^; for mouse primer/probe sequences, see Table 3). All primer sets were designed to amplify across an intron-exon boundary to avoid nuclear DNA amplification. Each primer was tested using a serial dilution series of cDNA from rat or mouse whole brain and SsoFast EvaGreen Supermix (BioRad), followed by melt curve analysis to verify amplification of one product. The resulting amplification mixture was tested by 4% agarose gel electrophoresis to confirm that the size of the amplified cDNA fragment matched the designed amplicon size. Once primers were verified, each primer set was optimized to 90–95% amplification efficiency using probes specific to the amplified fragment and iQ Supermix (BioRad).

### Pre-amplification (Multiplex) Reaction

For all rat and mouse single cell experiments, a pre-amplification reaction was performed in order to increase starting cDNA levels and avoid false negatives. For multiplex reactions, primers were grouped and tested with cDNA template of 10 ng/μL from whole brain homogenate to ensure that no primer cross binding or any other primer interaction occurred that would interfere with cDNA amplification in the multiplex reaction. Next, every cell was divided into three equal portions of approximately 4 μL each. Each primer was assigned into one of three groups, after which a mixture including iQ Supermix (BioRad), ddH_2_O, and one group of 10-fold diluted primers was added to each aliquot. All cells were tested using the same species-specific primer groups. All aliquots were then placed in a C1000 Thermocycler (BioRad) and processed as follows: 95 °C hot start for 3 minutes, followed by 15 cycles of 95 °C for 15 seconds, 57 °C for 20 seconds, and 72 °C for 25 seconds.

### Quantitative PCR Reaction

For qPCR, cDNA from the pre-amplified multiplex reaction was used for probe-based gene detection. Each target was run individually in triplicate, with undiluted primers, the appropriate FAM-TAMRA probe (Applied BioSystems, Inc.) specific to each target and species, and iQ Supermix (BioRad). Each cell was run on a CFX96 qPCR machine (BioRad) according to the following protocol: 95 °C hot start for three minutes, followed by 60 cycles of 95 °C for 15 seconds, 57 °C for 20 seconds, and 72 °C for 25 seconds. Amplification was measured by increased relative fluorescence during each cycle and a cycle threshold (Ct) value was assigned to each target using BioRad CFX Manager software. Proper amplification of each cellular target was also examined using 4% agarose gel electrophoresis to verify amplicon size.

### Data Analysis

Ct value data from the qPCR reaction from each cell was compared to Ct data from artificial cerebrospinal fluid samples extracted from each slice. If any target noted in artificial cerebrospinal fluid samples was within 5 cycles of the cell Ct value, it was excluded from the cell analysis, on a target-by-target basis. In a few cases, artificial cerebrospinal fluid samples displayed expression of targets; in this case, the cells taken from the slices corresponding to the artificial cerebrospinal fluid sample were classified as failures and not further analyzed. Ct values for 18S were subtracted from the Ct value for each target in a cell-wise manner to obtain a ΔCt value for each target. Any target with a ΔCt value greater than 20 was excluded from analysis as non-specific. To quantify mRNA expression levels, the ΔΔCt method^89^ was used. Relative expression was normalized to DA cells. Expression data was obtained using CFX Manager software (BioRad). All expression levels were tested for significance using an unpaired two-way Student’s T-test.

### Immunohistochemistry

Mouse GAD67-GFP brains were either transcardially perfused with 0.1 M phosphate-buffered saline (PBS) followed by 4% paraformaldehyde in 0.1 M PBS (pH 7.4) or rapidly dissected and fixed overnight in 4% paraformaldehyde in 0.1 M PBS (pH 7.4). Brains were cryoprotected in 30% sucrose solution, frozen in OCT, sliced into 30 μm sections and collected into 0.1 M PBS for a free-floating staining procedure. Slices were permeablized with 0.2% Triton-X (Fisher Bioreagents) for 30 minutes, washed with 1% bovine serum albumin in 0.1 M PBS for 2 hours, and treated with primary antibody for sheep anti-TH (1:500, Novus Biologicals), rabbit anti-NAPE-PLD (1:500, kindly provided by Dr. Ken Mackie), rabbit anti-DAGLα (1:1000, kindly provided by Dr. Ken Mackie), or rabbit anti-mGluR5 (1:500, Abcam) in 5% normal goat serum and 1% bovine serum albumin in PBS overnight at 10 °C. Slices were then washed twice with 0.1 M PBS, followed by one wash of 0.2% Triton-X (Fisher Bioreagents) in 0.1 M PBS for 30 minutes, one wash of 1% bovine serum albumin and 5% normal goat serum in 0.1 M PBS for 2 hours, and a final wash of anti-rabbit (1:1000, AlexaFluor 350, Invitrogen) or anti-sheep (1:500, AlexaFluor 546, Invitrogen) secondary antibody in 5% normal goat serum, 1% bovine serum albumin in PBS for 2 hours at room temperature. Slices were washed three times with tris-buffered saline and mounted onto Superfrost Plus microscope slides (VWR). For triple-label experiments, slices were washed for 10 minutes with 5% hydrogen peroxide and 5% methanol in PBS between each primary/secondary antibody pair. After drying overnight, slides were coverslipped with Fluoromount G (Southern Biotech) and imaged on an Olympus FluoView FV1000 laser scanning confocal microscope. Image capture was performed by sequential excitation of each fluorophore. Each 20×-magnified image was divided into nine equal sections for semi-quantitative analysis (analysis performed by two individuals independently counting visually labeled cells rather quantifying based on pixel analysis). Positively labeled GAD67-GFP, single immunolabeled, and double immunolabeled cells were visually identified, counted in each section, and totaled. Positive double-labeled cells are expressed as a percentage of total GAD67-GFP-labeled cells. Semi-quantitative percentages were compared to mRNA expression percentages from RT-qPCR experiments in order to compare mRNA expression and protein expression detected using immunohistochemistry.

## Additional Information

**How to cite this article**: Merrill, C. B. *et al.* Ventral tegmental area dopamine and GABA neurons: Physiological properties and expression of mRNA for endocannabinoid biosynthetic elements. *Sci. Rep.* **5**, 16176; doi: 10.1038/srep16176 (2015).

## Figures and Tables

**Table 1:** Calcium-binding protein and neuropeptide mRNA expression within rat ventral tegmental area neurons.

Calcium-binding protein expression	DA	GABA
PV	18.8%	58.3%
CB	12.5%	66.7%
CR	16.3%	58.3%
CCK	43.8%	50.0%
Co-expression in rat ventral tegmental area neurons		
PV, CB, CR, CCK	0%	25.0%
PV, CB, CCK	0%	16.7%
PV, CB	6.3%	0%
CB, CCK	6.3%	8.3%
CR only	6.3%	25.0%

**Table 2:** Number of cells expressing mRNA in rat and mouse VTA neurons for the following.

Cell type	DAGLα	NAPE-PLD	12LO	mGluR1	mGluR5
Rat DA (n = 16)	7 (43.8%)	6 (37.5%)	2 (12.5%)	3 (18.8%)	5 (31.3%)
Rat GABA (n = 12)	7 (58.3%)	2 (16.7%)	2 (16.7%)	4 (33.3%)	7 (58.3%)
Mouse GABA (n = 12)	5 (41.7%)	1 (8.3%)	4 (33.3%)	5 (41.7%)	5 (41.7%)
Co-expression of eCB biosynthetic enzyme and type I mGluR mRNA in VTA Neurons.					

	mGluR1/DAGLα	mGluR1/NAPE-PLD	mGluR1/12LO
Rat DA	X	X	
Rat GABA	X	X	X
Mouse GABA	X	X	X
	mGluR5/DAGLα	mGluR5/NAPE-PLD	mGluR5/12LO
Rat DA	X	X	X
Rat GABA	X	X	X
Mouse GABA	X	X	X

Note: In the bottom panel of the table, X indicates co-expression in one or more cells.

**Table 3:** Mouse Primer and Probe Sequences (bp, base pairs).

Target	Direction	Primer Sequence	Probe Sequence
**18S**	Forward	GTGCATGGCCGTTCTTAGTTG	TGGAGCGATTTGTCTGGTTAATTCCGATAAC
133 bp	Reverse	GCCACTTGTCCCTCTAAGAAGTTG	
**mGluR1**	Forward	ATATCGTCAAGCGGTACAACTG	TGCAGTCCACACAGAAGGGAATTACGG
102 bp	Reverse	GGCAGCCAACTCTTTGAAAG	
**mGluR5**	Forward	CTGCACACCTTGTAAGGAGAATG	TACACCTGCAAGGCGTGCCAACTG
103 bp	Reverse	CAAATCACAACCTGTCAAGTCG	
**DAGLα**	Forward	AGAAGAAGTTGGAGCAGGAGATG	ACCTGGGCCGTGGAACCAAACACTA
100 bp	Reverse	AAGGAGTGGCCTACCACAATC	
**NAPE-PLD**	Forward	CTGGACTGCATCCTCAAACG	AGCTAGCCCTCGGGATCAACAGCG
114 bp	Reverse	CAACGTCCGCTTGCTGTAC	
**12LO**	Forward	GCCAAGAGAAGCAGCAAGATG	AAGACTCGCTCTCAGATGCCCTACAAAGTG
100 bp	Reverse	CATCCTCAGTCCCAGAAAAGTG	
**GAD67**	Forward	ATCATGGCTGCTCGTTACAAGTAC	CATGGCGGCTGTGCCCAAACT
100 bp	Reverse	AATAGTGACTGTGTTCTGAGGTGAAG	
**TH**	Forward	GGACAAGCTCAGGAACTA	TCTCGTATCCAGCGCCCATTCTC
67 bp	Reverse	GGTGTACGGGTCAAACTTC	
**DAT**	Forward	AACCTGTACTGGCGGCTATG	CCCCTGCTTCCTTCTGTATGTGGTCG
87 bp	Reverse	GGGTCTGAAGGTCACAATGC	
